# Magnetic Seizure Therapy in Treatment-Resistant Schizophrenia: A Pilot Study

**DOI:** 10.3389/fpsyt.2017.00310

**Published:** 2018-01-16

**Authors:** Victor M. Tang, Daniel M. Blumberger, Shawn M. McClintock, Tyler S. Kaster, Tarek K. Rajji, Jonathan Downar, Paul B. Fitzgerald, Zafiris J. Daskalakis

**Affiliations:** ^1^Department of Psychiatry, University of Toronto, Toronto, ON, Canada; ^2^Temerty Centre for Therapeutic Brain Intervention, Centre for Addiction and Mental Health, Toronto, ON, Canada; ^3^Campbell Family Mental Health Research Institute, Centre for Addiction and Mental Health, University of Toronto, Toronto, ON, Canada; ^4^Faculty of Medicine, Institute of Medical Science, University of Toronto, Toronto, ON, Canada; ^5^Department of Psychiatry, UT Southwestern Medical Center, Dallas, TX, United States; ^6^Department of Psychiatry and Behavioral Sciences, Duke University School of Medicine, Durham, NC, United States; ^7^Krembil Research Institute, University Health Network, Toronto, ON, Canada; ^8^MRI-Guided rTMS Clinic, University Health Network, Toronto, ON, Canada; ^9^Monash Alfred Psychiatry Research Centre, The Alfred and Monash University Central Clinical School, Melbourne, VIC, Australia

**Keywords:** magnetic seizure therapy, electroconvulsive therapy, cognition, schizophrenia, schizoaffective disorder, brain stimulation, neuromodulation

## Abstract

**Objective:**

Electroconvulsive therapy is effective in treatment-resistant schizophrenia (TRS) but use is limited due to stigma and concerns around cognitive adverse effects. Magnetic seizure therapy (MST) is a promising new neuromodulation technique that uses transcranial magnetic stimulation to induce therapeutic seizures. Studies of MST in depression have shown clinical improvement with a favorable adverse effect profile. No studies have examined the clinical utility of MST in schizophrenia.

**Methods:**

We conducted an open-label pilot clinical trial of MST in eight TRS patients. Up to 24 MST treatments were delivered depending on treatment response. We assessed clinical outcome through the Brief Psychiatric Rating Scale (BPRS) and the Quality of Life Enjoyment and Satisfaction Questionnaire (Q-LES-Q). Cognitive testing included a neuropsychological test battery, the Autobiographical Memory Inventory (AMI), Montreal Cognitive Assessment (MoCA), and reorientation time.

**Results:**

Four patients completed the trial as per protocol. For all patients and for trial completers alone, there was a significant clinical and quality of life improvement. Three met pre-determined criteria for remission (total score ≤25 on the BPRS) and one met criteria for response (i.e., ≥25% BPRS improvement from baseline for two consecutive assessments). Pre and post neurocognitive data showed no significant cognitive adverse effects apart from a decrease in AMI scores.

**Conclusion:**

In this pilot study, MST demonstrated evidence for feasibility in patients with TRS, with promise for clinical efficacy and negligible cognitive side effects. Further study in larger clinical populations is needed.

**Clinical Trial Registration:**

www.ClinicalTrials.gov, Identifier NCT01596608.

## Introduction

Schizophrenia is a severe psychiatric disorder that causes a substantial burden to patients, families, and society as a whole (1). Unfortunately, an estimated 40% of patients with schizophrenia attain only a partial response and in 10% show little to no response (2). The condition of non-response is referred to as treatment-resistant schizophrenia (TRS), commonly defined in the literature as failure of two or more adequate antipsychotic trials and continued clinical and functional impairment (3). At present, the only treatment with a specific indication for TRS is clozapine (2). However, clozapine use is limited due to its side effects and the requirement of frequent monitoring of blood work (4). Further, research has found that approximately 25% of TRS patients also do not respond to treatment with clozapine (5).

Electroconvulsive therapy (ECT) is safe and effective in combination with standard antipsychotic therapy (6, 7), and particularly in cases of treatment resistance (8). Recently, ECT has demonstrated efficacy in augmenting clozapine response for TRS patients in a randomized controlled clinical trial (9). However, there are major limitations to the use of ECT, including the commonly associated cognitive adverse effects that include executive dysfunction, amnesia, and disorientation (10). Although these cognitive effects are now dramatically reduced using modern stimulus parameters (11), they remain a feared complication amongst patients (12). This, in combination with the considerable stigma surrounding ECT (13), prevents widespread acceptance of ECT among patients with schizophrenia.

Magnetic seizure therapy (MST) is a newer neuromodulation treatment that induces therapeutic seizures through the use of high-frequency repetitive transcranial magnetic stimulation (14, 15). Electrical current is generated from a magnetic pulse passed into the brain to provide directed, focal stimulation. As a result, MST avoids direct stimulation of the deep medial temporal lobe structures thought to be related to the cognitive adverse effects (16). In contrast, ECT uses an electrical stimulation that is impeded by the scalp and skull, with field-modeling studies suggesting widespread stimulation of cortical and subcortical regions including the medial temporal lobe (17). Thus, MST has been proposed as a possible alternative to ECT that could spare medial temporal structures from stimulation, and may have a favorable clinical benefit to cognitive side-effect risk profile. As a novel treatment modality, it has the potential to be free of the heavy burden of stigma that has long been associated with ECT. Clinical studies that have compared MST to unilateral (UL) or bilateral ECT in major depressive disorder (MDD) to date have demonstrated it is associated with shorter reorientation times after treatment (18) and fewer cognitive adverse effects (14, 19). However, one study found that neither right UL ECT nor MST produced significant cognitive side effects (20). Another recent open label study found a benign cognitive profile after MST in MDD patients (21). Additionally, MST has been shown to be associated with a significant reduction of suicidal ideation in patients with treatment-resistant depression (15), which may be a useful feature in TRS as schizophrenia is associated with an increased risk of suicide (1). These findings support the possibility that MST could offer a relatively cognition-sparing and clinically useful alternative to ECT for treatment-resistant illnesses other than MDD.

The purpose of this pilot study was to assess the clinical and cognitive effects of MST in patients with TRS. To our knowledge, this is the first report of MST use in patients with schizophrenia. As ECT has been found to be effective in schizophrenia (6, 9), we hypothesized that MST would also be effective in improving symptom severity and quality of life, while having a benign cognitive profile.

## Materials and Methods

### Participants

The study protocol was approved by the Institutional Research Ethics Board at the Centre for Addiction and Mental Health, a tertiary mental health facility associated with the University of Toronto. All patients provided written informed consent, and no financial compensation was provided. Patients were recruited from an ongoing open label MST clinical trial in multiple serious mental illnesses (NCT01596608). Patients with a diagnosis of schizophrenia or schizoaffective disorder as assessed by the structured clinical interview for the Diagnostic and Statistical Manual of Mental Disorders fourth version (SCID-IV) (22) were included in the study. Eligibility for the study required age range within 18–85, a moderate to severe symptom burden as assessed with the Brief Psychiatric Rating Scale (BPRS) (23) score of ≥37, and the capacity to give informed consent according to study and treating psychiatrist. Treatment resistance was quantified with the Antidepressant Treatment History Form (ATHF), modified for antipsychotic treatment trials (24). In the modified ATHF, each antipsychotic medication trial was rated for adequacy on a scale of 1–3. Any trial less than 3 weeks was rated at 1. If greater than or equal to 3 weeks, each antipsychotic is rated 1–3 for low, moderate, or high dose. The dose equivalency table has been reported elsewhere (24). We reported the cumulative score for the current episode of illness. No pre-determined criteria for treatment resistance were required for inclusion. Patients referred for our ECT or MST service are typically thought to be treatment resistant by their referring physician. All patients assessed for the study had either failed two adequate antipsychotic trials or clozapine for their current episode.

Exclusion criteria included: patients with unstable medical and/or neurological conditions, currently pregnant or lactating, not considered safe to undergo anesthesia, having any metallic implants in the head, cardiac pacemakers, cochlear implants, or other implanted electronic devices, taking a benzodiazepine at a dose greater than the equivalent of 2 mg of lorazepam, taking any non-benzodiazepine anticonvulsant, active substance abuse in the last 3 months, carry a current diagnosis of delirium, dementia, or cognitive disorder secondary to a general medical condition, other significant Diagnostic and Statistical Manual of Mental Disorders-IV (DSM-IV) Axis I comorbidity, the presence of borderline personality disorder or antisocial personality disorder as assessed by the Structured Clinical Interview for DSM-IV Axis II disorders (SCID-II), and any suicide attempts within the last 6 months.

### MST Treatment

Magnetic seizure therapy treatments were provided under general anesthesia using methohexital sodium (0.375–0.75 mg/kg IV) and neuromuscular blockade with succinylcholine chloride (0.5–1.0 mg/kg IV). If a patient was determined by a trained study psychiatrist to be having inadequate seizures, the dose of methohexital was decreased and remifentanil (1.0–1.5 µg/kg) was added as a second anesthetic agent as used in convulsive therapy practice. Blood pressure, oxygen saturation, heart rate, and EKG were monitored throughout the entire MST treatment procedure. A MagVenture twin coil (comprised of two circular coils) was placed on the frontal cortex symmetrically over F3 and F4 according to the international 10–20 system, producing a maximum electric field in the midline between the two circular coils. Stimulation trains were provided at 100% machine output at frequency of 25–100 Hz from a stimulator machine (MagPro MST; MagVenture). Stimulus frequency was fixed throughout the duration of treatment and determined before the start of the treatment. Different stimulus frequencies were used over the course of the trial period. Seizure threshold was determined at 100% stimulator output with the selected treatment frequency, and train durations were escalated until an adequate seizure was produced. Stimulus duration was initially set at 4 s for 25 Hz settings and 2 s for 50–100 Hz. During initial titration, a maximum of three stimulations were provided within the same first session, with increments each time of 8 s for 25 Hz and 4 s for 50–100 Hz settings. The maximum stimulus duration was 1,000 pulses. For treatment after titration, stimulus was set higher than the threshold stimulus at 8 s for 25 Hz and 4 s for 50–100 Hz. If an adequate seizure was not achieved, the titration would be continued at the next session. The adequacy of the seizure was determined at each session by the treating MST psychiatrist (e.g., generalized tonic-clonic seizures that are typically less than 15 s in duration). Sessions were delivered 2–3 times per week. The treatment course of MST was a maximum of 24 sessions or until the patient achieved remission.

### Clinical Assessment

Demographic measures were collected at baseline. Symptoms of schizophrenia were measured using the BPRS, and assessments were performed at baseline and after every three MST sessions. Patients who showed no improvement by 30% on the BPRS total score from the last assessment had an increase in dose by stimulation duration for their next treatment. Specifically, for 25 Hz settings, duration was increased 4 s, and for 50 or 100 Hz by 2 s. The primary outcome variable was change in the BPRS total score from pre- to posttreatment course. Response criterion at the end of the treatment course was defined as having ≥25% BPRS improvement from baseline for two consecutive assessments. Remission was defined as a total score ≤25 on the BPRS, which indicates mild schizophrenia symptom burden. Secondary outcomes included impact on quality of life, assessed with the Quality of Life Enjoyment and Satisfaction Questionnaire (Q-LES-Q) (25) prior to and at the end of the treatment phase, and cognitive functioning.

### Cognitive Assessments

Patients completed a comprehensive neurocognitive test battery prior to and upon completion of the treatment phase. The neurocognitive battery included the Autobiographical Memory Inventory (AMI) short form (26), the MATRICS Consensus Cognitive Battery (MCCB; excluding the Mayer-Salovey-Caruso Emotional Intelligence Test and Continuous Performance Test—Identical Pairs) (27), Trail Making Test (TMT): Part B (28), Stroop Test (29) and Verbal Fluency using the Controlled Oral Word Association Test (COWAT) (30). The MCCB is a standardized neurocognitive battery for the assessment of adults with schizophrenia. In addition, we administered the Montreal Cognitive Assessment (MoCA) at baseline and after every six treatments, and assessed general intellectual ability with the Wechsler Test of Adult Reading (31) at baseline. Time to reorientation was measured after each MST session. Reorientation was defined as correct response to personal name, date of birth, age, place, and day of the week.

### Data Analysis

The BPRS, Q-LES-Q, and cognitive assessments were compared between baseline and posttreatment using paired *t*-tests. For BPRS total scores, one analysis was computed only with the data of patients who completed the treatment course (completers), and another analysis was computed with all patients with a last observation carried forward intent-to-treat analysis to account for missing data. Only the completers and one patient who withdrew before his last treatment were analyzed for the Q-LES-Q, as this was only assessed at the beginning and end of the treatment course. An analysis was computed for all neurocognitive measures including reorientation time for the completers and the one patient who withdrew before his last treatment. For reorientation time and the MoCA, another intent-to-treat analysis with last observation carried forward was computed for all patients. Statistical significance was set as *P* ≤ 0.05.

## Results

### Patient Demographics and Flow through Study

A total of eight patients (mean age = 45.88 ± 12.31) were enrolled in the study and received MST treatments. Seven of the patients were male, the average duration of schizophrenia illness was 24.88 years (SD = 10.72), and all patients met criteria for TRS (see Table 1). None of the patients currently met the criteria for a major depressive episode. For three patients, psychotropic medications were adjusted within the MST treatments (as detailed in Table 1). All patients either met criteria for treatment resistance in the current episode (i.e., having failed over two adequate trials of antipsychotic medications or >6 on the ATHF cumulative score for current episode) or were currently on a failed trial of clozapine, a medication that is only indicated in treatment resistance schizophrenia. The average ATHF cumulative score was 12.50 (SD = 8.14).

**Table 1:** Patient demographic information and illness history.

Case	Age range	Illness duration (years)	Diagnosis	ATHF (cumulative score)	Baseline meds	Med changes
1	31–35	16	SCZ	3	Clozapine, Clonzepam	No change
2	56–59	44	SCZ	8	Risperidone, Clonazepam, Venlafaxine	No change
3	51–55	39	SCZ	16	Lorazepam, Atorvastatin, Levothyroxine, Olanzapine	No change
4	51–55	25	SCZ-A	18	Lithium, Lorazepam, Lamotrigine, Valproic acid, Quetiapine, Pantoprazole, Risperidone, Levothyroxine	T3: D/C Lithium, Lorazepam, Lamotrigine, Valproic Acid
5	41–45	23	SCZ	26	Lurasidone	T1: Added Amisulpride, Zopiclone, D/C Lurasidone
6	26–30	11	SCZ	5	Clozapine, Paliperidone, Valproic acid	T3: D/C Valproic acid
7	36–40	16	SCZ	18	Clozapine, Escitalopram, Atenolol, Rosuvastatin, Metformin, Pantoprazole, Sennosides, Clonazepam	T23: Decreased escitalopram
8	60–65	25	SCZ-A	6	Clozapine, Vitamin B Complex, Calcium Carbonate, Vitamin D, Clopidogrel, Coenzyme Q10, Dexlansoprazole, Multivitamin, Levothyroxine, Linaclotide, Magnesium citrate, Alirocumab	T18: Increased dexlansoprazole

*SCZ, schizophrenia; SCZ-A, schizoaffective; ATHF, antidepressant treatment history form (modified for antipsychotics), cumulative score for current episode; medication changes at time of treatment (for example, T3 = Treatment 3). D/C, discontinued*.

**Figure 1:**
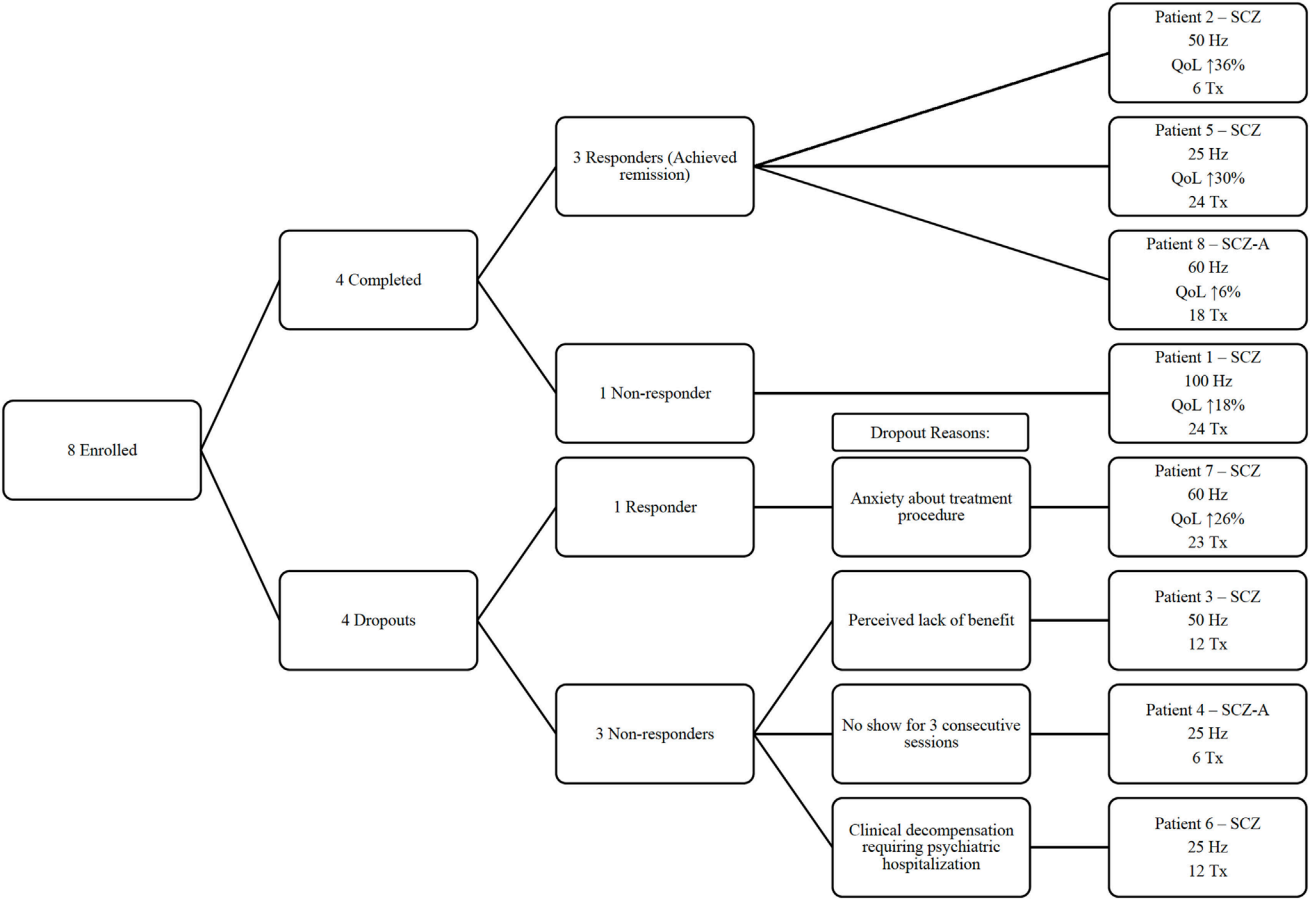
Flow diagram for individual patients enrolled in the study. SCZ, schizophrenia; SCZ-A, schizoaffective. QoL, quality of life as measured by the Quality of Life Enjoyment and Satisfaction Questionnaire; Hz, frequency of stimulation; Tx, number of magnetic seizure therapy treatment sessions completed.

Four patients did not complete treatment. For the patients who did not complete treatment, Patient 3 withdrew after 12 treatments due to perceived lack of benefit, and Patient 4 was discontinued after six treatments for missing the next three consecutive treatment sessions. Patient 6 was discontinued from the study after a clinical decompensation that required an involuntary hospitalization after 12 treatments. Patient 7 withdrew one session short of the full treatment course (i.e., 24 treatments), as he grew increasingly anxious about the treatment procedure. A flow diagram in Figure 1 highlights the main outcomes of each individual patient.

### Clinical and Quality of Life Outcome

Table 2 shows individual clinical outcomes from the course of MST. Of the eight patients, three (Patient 2, 5, 8) reached remission criteria and one patient (Patient 7) reached response criteria. Among completers of the MST treatment protocol, three remitted, and one had no response. In non-completers, 1 responded and three had no response. Of the remitters, Patients 2 and 5 had a substantial improvement in quality of life, with a 30 and 36% improvement in overall scores, respectively, and Patient 8 only had a 6% improvement. Patient 7 was a responder and had a 26% improvement in the Q-LES-Q total score. In contrast, Patient 1 was a non-responder and showed an 18% improvement in the Q-LES-Q.

**Table 2:** Clinical response characteristics for individual patients.

Patient	Completed (Y/N)	# of treatments	Time to response	Baseline BPRS	Post BPRS	BPRS % improvement	Baseline QoL	Post QoL	QoL % improvement	Frequency (Hz)
1	Y	24	NR	40	32	20%	40	58	18%	100
2	Y	6	3	40	22	45%	52	88	36%	50
3	N	12	NR	44	36	18%	52	–	–	50
4	N	6	NR	38	43	−13%	61	–	–	25
5	Y	24	9	42	24	43%	38	68	30%	25
6	N	12	NR	45	46	−2%	11	–	–	25
7	N	23	6	52	32	38%	16	36	26%	60
8	Y	18	9	40	24	40%	48	54	6%	60

*NR, no response; BPRS, Brief Psychiatric Rating Scale; QoL, quality of life assessed by Quality of Life Enjoyment and Satisfaction Questionnaire Short Form (Q-LES-Q SF), reported as percentage of maximum possible score; frequency, magnetic seizure therapy stimulation used to achieve therapeutic seizures*.

In a last observation carried forward analysis (Table 3), the group mean BPRS total score showed a statistically significant reduction from baseline (mean = 42.63, SD = 4.44) to end [mean = 32.38, SD = 8.94; *t*(7) = 3.087; *P* = 0.018]. Completers analysis of BPRS scores (Table 3) showed a significant mean improvement from 40.50 ± 1.00 to 25.50 ± 4.43 [*t*(3) = 6.301; *P* = 0.008]. Cohen’s *d* for the total group difference and the completers group difference was 1.452 and 4.671, respectively, suggesting a large effect size difference for BPRS improvement from MST. In the Q-LES-Q total score, there was a statistically significant increase of 38.80 ± 13.97 to 60.80 ± 19.11 [*t*(4) = −4.250; *P* = 0.013]. For Q-LES-Q scores, Cohen’s *d* = −1.314 suggesting large effect size.

**Table 3:** Clinical outcomes group analysis.

		Pre treatment		Post treatment			
	*n*	Mean	SD	Mean	SD	*P*	Cohen’s *d*
Completers BPRS	4	40.50	1.00	25.50	4.43	0.008	4.671
Q-LES-Q	5	38.80	13.97	60.80	19.11	0.013	–1.314
All subjects BPRS	8	42.63	4.44	32.38	8.94	0.018	1.452

*BPRS, Brief Psychiatric Rating Scale; Q-LES-Q, Quality of Life Enjoyment and Satisfaction Questionnaire Short Form*.

### Cognitive Outcome

There were no significant differences on the TMT Part B, COWAT, or any MCCB domain score between pre and posttreatment in completers (Table 4). There were no significant differences on the MoCA [mean difference 1.83 ± 2.137; *t*(5) = 2.101; *P* = 0.090] and reorientation time in minutes [mean difference 9.00 ± 16.254; *t*(7) = 1.566; *P* = 0.161] from baseline to last observation. The average reorientation time for the first MST session was 24.22 (SD = 22.70) minutes and for the last observed session 15.22 (SD = 12.77) minutes. There was a modest but statistically significant worsening from pre to posttreatment on the AMI-SF score [mean difference 9.8 ± 3.962; *t*(4) = 5.530; *P* = 0.005].

**Table 4:** Cognitive outcomes pre and post MST.

Cognitive domain	Measure	*P*-value	Mean change	SD	*n*
Autobiographical memory	AMI-SF	0.005	9.800	3.962	5
Speed of processing	BACS SC	0.755	1.200	8.043	5
	Fluency	0.471	3.800	10.686	5
	TMT-A	0.243	6.400	10.455	5
Working memory nonverbal	Spatial span	0.177	5.800	7.918	5
Working memory verbal	LNS	0.882	0.750	9.287	4
Verbal learning	HVLT-R	0.521	2.600	8.264	5
Visual learning	BVMT-R	0.607	2.800	11.234	5
Reasoning and problem solving	Mazes	0.220	4.800	7.396	5
Cognitive set-shifting	TMT-B	0.236	15.000	15.524	3
Processing speed and inhibition	Stroop	0.263	12.400	21.279	5
Verbal fluency	COWAT	0.105	8.400	8.989	5
Mild cognitive impairment	MoCA	0.090	1.833	2.136	5

*Mean change, post- minus pre- magnetic seizure therapy scores*.

*AMI-SF, Autobiographical Memory Inventory Short Form; BACS SC, Brief Assessment of Cognition in Schizophrenia Symbol Coding; Fluency, category fluency: animal naming; TMT-A, Trail Making Test Part A; Spatial Span from the Weschler Memory Scale—third edition; LNS, letter-number span; HVLT-R, Hopkins Verbal Learning Test—Revised; BVMT-R, Brief Visuospatial Memory Test—Revised; Mazes from Neuropsychological Assessment Battery; TMT-B, Trail Making Test Part B; Stroop, Stroop Color-Word Test; COWAT, Controlled Oral Word Association Test; MoCA, Montreal Cognitive Assessment*.

## Discussion

The results of this pilot study suggest that MST may be an efficacious treatment in patients with TRS. Of the eight patients treated with MST, there was a significant improvement in BPRS scores and Q-LES-Q scores posttreatment compared to pretreatment. Half of the patients had a clinically significant response to treatment at the end of the treatment phase, with three patients achieving symptom remission. Remission was pre-determined to be equal or less than 25 overall on the BPRS, which indicates a very mild symptom burden remaining. Indeed, a score of 31 on the BPRS has been linked to global clinical impression ratings of “mildly ill” (32). However, four patients discontinued treatment before completing the full course of planned sessions. Patient 5 had a new medication around the time of treatment initiation. However, this is unlikely to have contributed significantly to the patients improvement given that this patient started the trial with an ATHF score of 26. Since three points on the ATHF represents a single adequate treatment trial that has failed, this patient had failed or could not tolerate numerous antipsychotic medications—including clozapine. A recent meta-analysis of RCTs that examined the combination of ECT with non-clozapine antipsychotics found a response rate of 50.9% compared to 32.9% in non-clozapine antipsychotic monotherapy (33). For patients who are clozapine-resistant, a recent meta-analysis of four open-label clinical trials and one randomized single blinded RCT in patients inadequately responding to clozapine reported a pooled response proportion of 54% with combination treatment of clozapine and ECT treatment (34). This meta-analysis includes the first prospective randomized study of ECT in clozapine-resistant schizophrenia patients, where 50% of patients responded (9). Controlled trials directly comparing ECT to MST in a larger sample are required to determine if rates of response are similar between the two treatment modalities.

We examined the effects of MST on multiple cognitive domains (Table 4). For those patients who completed the treatment course, no significant differences were found before and after treatment of MST in any of the cognitive tests in the MCCB, Stroop test, or MoCA for completers. There was no significant group difference between baseline reorientation time and last observation among all subjects. These results suggest that a completed treatment course of MST may have no observable adverse cognitive effects. Importantly, the MCCB is a standard for assessing cognitive function specifically for patients with schizophrenia and our findings show a lack of deterioration in all MCCB domains measured. This is in line with studies of MST for depression, which have suggested a lack of significant differences in cognitive measurements before and after MST treatment (21), and less cognitive side effects when compared with ECT (16).

However, consistency of autobiographical recall was the only cognitive domain that showed a significant worsening from pre to posttreatment. While this may suggest that there are MST associated adverse effects on autobiographical memory consistency, it is possible that the effect was more associated with time rather than only with treatment. Indeed, the psychometric properties of the AMI-SF have been called into question (35, 36) as it measures consistency in autobiographical recall rather than the presence or absence of specific autobiographical memories *per se*. Consistency of memory recall can be expected to decrease with the passage of time, as healthy adults tend to show on average between a 28 and 40% decrease on autobiographical memory consistency over a span of 1 week to 3 months (35). As the posttreatment AMI-SF score is derived from the ability of the patient to provide the identical details from the pretreatment responses, the score can only remain stable or decline, it can never show improvement. Lastly, it is unlikely that MST would result in a specific deficit in the AMI without affecting other cognitive domains, particularly those that are not affected by time, as was observed in these results. Future studies should include a control group in order to determine if the change on the AMI-SF was an effect of time, MST, or an interaction of both.

Of note, none of the reasons for discontinuing treatment in the four non-completers included cognitive adverse effects. Furthermore, in the intent-to-treat analysis when all patients were assessed for differences between baseline and last observed reorientation time and MoCA scores, the difference did not reach significance. Larger scale reviews on adverse effects in ECT have demonstrated a robust negative impact on neurocognitive function (10, 11). Of the reports from available clinical trials in schizophrenia, ECT combined with clozapine resulted in decreased processing speed and verbal learning relative to clozapine alone (9), and before and after an acute ECT course, there was little to no change on global cognitive function (37). In an open-label study of eight patients, two had subjective complaints of memory impairments during the course of ECT (38).

A significant limitation to the study was the small sample size, particularly of those that completed the entire course of treatment. The current study was also open label without a comparator group. The large effect sizes on clinical improvement from our results may be an over-estimation of the true effect due to the open-label method of the study. The high dropout rate suggests the difficulty of treating patients with TRS, particularly for treatment modalities that require frequent visits over a long period of time. A standard MST or ECT protocol requires that patients be reliable enough to attend 2–3 appointments a week for 8–12 weeks. This difficulty becomes increased when considering the level of disorganization and severity of symptoms for those who suffer from TRS. Future studies of clinical trials in MST for TRS will require strategies to mitigate the problem of dropouts. Another limitation of our study is that only posttreatment effects were available and does not answer the important question of the long-term maintenance of effect when patients experience improvement. Lastly, the possible effect of anesthesia on treatment outcomes was not addressed in this study. In one study of 20 case matched patients, anesthetic procedures were compared between ECT and MST, and there was a decreased requirement for muscle relaxants in MST (39). Future study in this area is required.

## Conclusion

In summary, this preliminary study provides the first report supporting the use of MST in patients with schizophrenia. MST resulted in clinically meaningful improvements in half of the patients. No significant differences were found in most of the cognitive measures used, except for a change in consistency of autobiographical memory recall. To draw more meaningful conclusions about the effect on autobiographical memory, future studies will require a control group to control for the effect of time on autobiographical memory recall. The initial results from this sample are promising and warrant further study with MST as a novel treatment option for TRS.

## Ethics Statement

The study protocol was approved by the ethics committee at the Centre for Addiction and Mental Health, a tertiary mental health facility associated with the University of Toronto. All patients provided written informed consent, and no financial compensation was provided.

## Author Contributions

All authors (VT, DB, SM, TK, TR, JD, PF, and ZD) contributed to the conception, structure, and literature review for the manuscript. VT and ZD prepared the initial draft, and DB, SM, TK, TR, JD, and PF critically revised the draft. All authors (VT, DB, SM, TK, TR, JD, PF, and ZD) prepared the final draft. All authors (VT, DB, SM, TK, TR, JD, PF, and ZD) approved the final draft for publication and agreed to assume accountability for the accuracy and composition of the manuscript.

## Conflict of Interest Statement

Dr. Blumberger reports grants from Canadian Institutes of Health Research, grants from Brain Canada, grants from Brainsway, non-financial support from Magventure, outside the submitted work; Dr. McClintock reports grants from National Institutes of Health, personal fees from Pearson, outside the submitted work; Dr. Downar reports non-financial support from MagVenture, non-financial support from ANT Neuro, outside the submitted work; Dr. Fitzgerald reports other from Magventure, other from Neuronetics, from Brainsway, personal fees from LivaNova, personal fees from Bionomics, outside the submitted work; Dr. Daskalakis reports philanthropic gifts from the Temerty Family Foundation and from the Grant Family Foundation, grants from Canadian Institute of Health Research, during the conduct of the study; grants from Brainsway, non-financial support from Magventure, advisory board from Sunovion, outside the submitted work.
